# Responsiveness of Physical Rehabilitation Centers in Capital of Iran: Disparities and Related Determinants in Public and Private Sectors

**DOI:** 10.3389/fpubh.2018.00317

**Published:** 2018-11-14

**Authors:** Manijeh Alavi, Mohammad Reza Khodaie Ardakani, Maziar Moradi-Lakeh, Homeira Sajjadi, Mohsen Shati, Mehdi Noroozi, Ameneh Setareh Forouzan

**Affiliations:** ^1^Social Determinants of Health Research Center, University of Social Welfare and Rehabilitation Sciences, Tehran, Iran; ^2^Research and Technology Department, Ministry of Health and Medical Education, Tehran, Iran; ^3^Department of Community and Family Medicine, Preventive Medicine and Public Health Research Center, Iran University of Medical Sciences, Tehran, Iran; ^4^Social Welfare Management Research Center, University of Social Welfare and Rehabilitation Sciences, Tehran, Iran; ^5^Department of Aging, University of Social Welfare and Rehabilitation Sciences, Tehran, Iran

**Keywords:** rehabilitation centers, health status disparities, inequality, responsiveness, Iran

## Abstract

**Background:** Responsiveness as a non-medical, non-financial goal of the health system is of special importance to people with physical disability. The current study assessed the experiences of people with physical disabilities when they encounter rehabilitation centers in Tehran.

**Methods:** This cross-sectional study was conducted in Tehran, the capital of Iran. The sample consisted of 610 people with physical disabilities referred to 10 comprehensive rehabilitation centers (CRCs) selected by Quota sampling. Data were collected by a standard responsiveness questionnaire proposed by the World Health Organization (WHO) and were analyzed by a standard protocol. Blinder-Oaxaca analysis was done to explain the inequality in performance of public and private sectors.

**Results:** Study participants included 298 (48.7%) women and 312 (51.3%) men. The mean age of the respondents was 46.3 (*SD* = 14.3) for women and 45.6 (*SD* = 15.4) for men. Prompt attention (33.3%) and confidentiality (1.3%) were the most and least important reported domains, respectively. Overall poor responsiveness was reported by 20.9% of respondents. Private rehabilitation centers showed significantly better performance in communication, basic amenities and autonomy compared to public centers (*P* ≤ 0.05). Perceived social class explained 76% of the inequality in autonomy in the private and public sector (*P* ≤ 0.05).

**Conclusion:** Improving overall responsiveness in domains that are of high importance from the respondents' viewpoint but are performing poorly—areas such as prompt attention and basic amenities—is essential. Additionally, interventions are needed to improve the performance of the public centers and providers in the areas of participation of service users in all social classes in their rehabilitation decisions and procedures, clear communication, and basic amenities.

## Introduction

Disability is one of the serious issues in the fields of medicine, rehabilitation, and social sciences, and its history dates back to the beginning of humanity (1). The United Nations, as well as many countries, consider disability an important topic on the health agenda (2). There are a billion people living with disabilities around the world, a figure that accounts for approximately 15% of the world's total population (1). People with disability are among the most vulnerable social groups, and they need special attention due to their situation. Physical disabilities, in turn, account for a significant proportion of all disabilities (3). More than 650 million people in the world suffer from physical disabilities, about two thirds of whom live in developing countries, including Iran (1). The World Health Organization (WHO) reported that people with disabilities are twice as likely to be faced with difficulties in access to health services, three times more likely to be neglected and four times more likely to be treated badly compared to people without disabilities (4). Despite the adoption of The Convention on the Rights of Persons with Disabilities, data collection and monitoring mechanisms in international development and global health still largely ignore those with disabilities (5).

Based on Iranian studies, more than 11 million of people in Iran are suffering from disabilities, most of whom are people with physical disabilities (3, 6). Road accidents, one of the main causes of mortality and morbidity in Iran, have had the biggest impact on increasing the rate of disabilities in Iran (7). Aging and chronic diseases are also increasing in Iran, subsequently causing increased rates of disability (8). Tehran, as the capital and the most populous city in Iran, with a population of over 12 million, accounts for the highest proportion of people with disabilities in the country (3).

This increasing prevalence of people with disabilities, in particular physical disabilities, requires continuous care that is mainly provided by health systems (2). The WHO proposes three explicit goals to assess the performance of a health system: improving health, fairness in financial contribution and responsiveness (9). Responsiveness as one of the intrinsic goals of a health system reflects how well that system is responding to the legitimate expectations of individuals regarding non-medical and non-financial issues (10, 11). The WHO Multi-country Survey Study in 2000–2001 produced valuable information about how health systems are responding to the legitimate expectations of populations in many countries around the world (12).

Responsiveness is considered of special importance, as it relates to human rights, has a positive relationship with health outcomes and can be successful achieved by low-cost interventions (13, 14).

Responsiveness is a multidimensional concept with 7 domains for out-patients, including autonomy (involvement in decisions related to health), choice (meeting with the health provider of one's own choice), communication (clarity of information received by the service user), confidentiality (privacy), dignity (respectful interaction), prompt attention (e.g., access, waiting times), and basic amenities (quality of basic facilities). Access to family and community support is only considered for inpatients. Today, autonomy has been globally noticed as a very important domain because service users' participation in decision-makings about their health is a main aspect of patient-centered care. It influences population health outcomes, improves quality and patients' safety and has an important role in patients' welfare and even containing health costs (15).

Responsiveness to people with disabilities becomes even more important, considering their large numbers in the population and their unmet needs in the field of health (16).

Although responsiveness has been studied in general hospitals (17–19) and in special outpatient populations, such as people with mental health disorders (11, 20–22), chronic disease (23), heart disease (24), diabetes (25) or, for inpatient, delivery care (26), there has been very little investigation into the experience of people with disabilities who receive rehabilitation services. Likewise, several studies indicate that there is a significant difference in responsiveness of public and private sector but there are few studies about the socio-economic characteristics that can explain this gap (19, 27–29).

The current study aimed to assess the experience of people with physical disabilities encountering rehabilitation centers in Tehran.

To achieve the objectives of this study, the following key questions were asked:

- How do people with physical disabilities assess rehabilitation service responsiveness?
- Which domains are the best and the worst performing?
- What are the most and least important domains for the service users?
- Is there any difference between the experiences of individuals who used public and private rehabilitation services?
- How do socio-demographic characteristics explain the gap between performance of public and private physical rehabilitation centers in domain of autonomy?

## Methods

The current study was a cross-sectional study carried out in comprehensive rehabilitation centers of Tehran, the capital city of Iran.

### Setting and selecting the comprehensive rehabilitation centers (CRCs)

In Iran, designing, planning, and implementing health policies and monitoring and supervising health-related activities in both public and private sectors are the responsibilities of the Ministry of Health and Medical Education (MOHME). Health policies are implemented and supervised through medical universities country-wide (30). Rehabilitation activities and services are mainly provided by public and private rehabilitation centers. All rehabilitation centers must be licensed by the medical universities, which act as the representative of MOHME. In 2016, there were 31 comprehensive rehabilitation centers (CRCs) licensed by three medical universities in Tehran. Comprehensive rehabilitation centers are affiliated with one of the organizations noted above and provide broad rehabilitation services, including physical, mental and social services.

Physical rehabilitation services include occupational therapy, physiotherapy, orthosis, prosthesis, etc. which are provided under the supervision of specialists in these fields.

### Selecting the comprehensive rehabilitation centers for the study

Eighteen out of 31 CRCs had licenses in the field of physical rehabilitation during the sampling period. Tehran was divided into five regions (North, South, Center, West, and East) based on division of the municipality. Quota sampling was used to select the centers for the study in order to have centers from public and private sectors as well as representing all regions of Tehran (North, South, Center, West and East) (22). Ten CRCs, including 5 public and 5 private, representing broad geographical coverage, were selected and agreed to participate in the study.

### The instrument

A standard questionnaire for responsiveness, proposed by WHO, was used to gather data. This questionnaire includes questions related to the use of the service, general health, and responsiveness. As the service users were in the outpatient setting, 7 domains of responsiveness, including prompt attention, dignity, choice, autonomy, confidentiality, clear communication, and basic amenities, were considered (questions are available as a Supplementary Material). The questionnaire was previously validated in Iran (14, 31). However, in the current study, internal consistency was examined using Cronbach's alpha. Kappa was also calculated by test-retest on 30 people. Cronbach's alpha of the 7 domains showed a range of at least 0.677 for prompt attention and a maximum of 0.911 for basic amenities. Kappa was at least 0.75 in prompt attention and a maximum of 0.94 in basic amenities. Missing rates for the 7 domains of the questionnaire were within 0.3–1%. Therefore, the WHO standard questionnaire for responsiveness was reliable and feasible to use in this population.

A demographic checklist was completed, including variables such as sex (male/ female based on self-recognition); age (self-reported in two groups as 18–59 and ≥60 years); education (self-reported in three groups as elementary with <5 years of education, intermediate with 5–12 years of education, and upper with >12 years of education); health assessment [self-reported in two groups as good health (very good/good) and bad health (moderate/bad/very bad)], social class [self-reported in three groups as low (very low/ low), middle, and high (very high/high)]. Physical disability was defined as musculoskeletal impairments that could be congenital, due to accidents or diseases, or other causes as specified by the respondent.

### Study population and sampling

A formula of the proportion estimation was used to calculate the sample size (32).

(1) $$\begin{array}{l} N=\;\frac{{\left[Z_{1-\;\frac{\propto }{2}}\right]}^{2}pq}{d^{2}} \end{array}$$

Where $Z_{1-\;\frac{\propto }{2}}$ is equal to 1.96. Also, “p” and “q” were considered based on previous studies on responsiveness in Iran (18, 30) and “d” was estimated as 0.15p. Finally, based on 5 geographical regions in Tehran the sample size was calculated and rounded as 610. The number of participants for each center was proportional according to the average number of monthly service users (based on a 3-month period). The final sample size by public and private centers was 406 and 204, respectively. People aged 18 years and over who were (1) diagnosed by a physician as having a physical disability, (2) referred to a selected center during the sampling period (from October 2016 through March 2017) and had experience using rehabilitation services in last 12 months, and (3) were mentally and physically capable to answer the questionnaire were included after informed written consent was obtained.

The questionnaire for each service user was completed by face to face interview. To minimize the social bias, two trained interviewers who were not staff members of the rehabilitation center along with the principal investigator, administered the questionnaires in a private area. The participants in the study were assured that their responses were completely confidential and had no effect on the process of receiving the rehabilitation services.

### Data analysis

Analysis of the data was conducted using the approach of the WHO analytical guideline for Multi-Country Survey (MCSS) (12). There were two to four questions to report experiences of service users and one “rating” question for each domain. The responsiveness score was calculated based on responses to the rating questions. Answers to a 5-point Likert scale were recoded using very good as (5) to very bad as (1). Performance of each domain was assessed as good if the response to the rating question of the domain was very good (5) or good (4) and as poor if the reply was moderate (3), bad (2) or very bad (1).

To determine the overall responsiveness, we summed the scores of each domain and averaged them, then categorized the scores into good (combining the very good and good) and poor (combining moderate, bad and very bad) responsiveness (22).

Based on distribution of data, means, and standard deviation were used to present central values and dispersions in case of symmetrical distribution and median was used if the distribution was asymmetrical.

Comparison of performance of public and private CRCs (as good and poor) was done by a chi-square test. Finally, to decompose the gap between good performance of autonomy in public and private physical rehabilitation centers Blinder-Oaxaca (BO) method was used (33, 34). Outcome of interest was good performance of autonomy domain by center type. The performance of autonomy variable was measured by a question: “Overall, how would you rate your experience of getting involved in making decision about your care or treatment (rehabilitation) as much as you wanted in the last 12 months.” The responses then dichotomized in to two groups as good autonomy (combining good and very good) and poor autonomy (combining moderate, bad, very bad).

Explanatory variables which included in the model were age (years), education (years), perceived health status (self-report as good health or bad health), perceived social class (self-report as low, middle, high), economic status [as residential area per capita (m^2^) -by calculating the ratio of residential area to household size-].

Blinder-Oaxaca decomposition model explains how much of the difference between the two groups in the outcome variables is due to differences in the explanatory variables included in the model, across the groups and how much is due to coefficient effect as well as the other characteristics that have not been included in the model (35).

(2) $$\begin{array}{l} {\bar{Y}}^{U}-{\bar{Y}}^{L}=\left[\overset{N^{L}}{\underset{i=1}{\sum }}\frac{F\left(X_{i}^{L}\beta^{H}\right)}{N^{L}}-\overset{N^{H}}{\underset{i=1}{\sum }}\frac{F\left(X_{i}^{H}\beta^{H}\right)}{N^{H}}\right]\\ \;+\left[\overset{N^{L}}{\underset{i=1}{\sum }}\frac{F\left(X_{i}^{L}\beta^{L}\right)}{N^{L}}-\overset{N^{L}}{\underset{i=1}{\sum }}\frac{F\left(X_{i}^{L}\beta^{H}\right)}{N^{L}}\right] \end{array}$$

In Equation (1), *N* refers to the sample size of public and private center users. In the first bracket, the phrase indicates the portion of the gap in the good performance in autonomy of public and private centers pertaining to differences in the explanatory characteristics that have been included in the model. The second phrase shows the part of the mentioned gap that relates to differences in the effects of these characteristics on the performance of autonomy (unexplained components or coefficient effect).

We used STATA software (V11) for the analysis. The level of statistical significance was considered as (*p* ≤ 0.05) in this study.

### Ethics and consent

This study was conducted after gaining approval from the ethical committee of the University of Social Welfare and Rehabilitation Sciences (ethical code: IR.USWR.REC.1395.86) and receiving permission from the management boards of the private and public rehabilitation centers and from the medical universities responsible for health services in the area. The service users who met the inclusion criteria were instructed about the goals of our study and were assured about confidentiality of data; they were included after providing informed written consent.

## Results

Of the 610 service users with physical disability included in our study, 298 (48.7%) were women and 312 (51.3%) were men. The mean age of the people referred to CRCs was 46.3 years (*SD* = 14.3) for women and 45.6 years (*SD* = 15.4) for men. All users had used only a single rehabilitation center during the past 12 months. Approximately one third of people using CRCs during last 12 months were the service users of private centers (34.3%), while public centers accounted for 65.7% of the sample.

Among various rehabilitation services, physiotherapy was the most commonly referred service (60.4%). Other services used were occupational therapy (32.1%), orthosis, and prosthesis (4.8%) and a mixture of physiotherapy and occupational therapy (2.7%).

The socio-demographic characteristics of the people with physical disability are shown in Table 1.

**Table 1:** Socio-demographic characteristics of the people with physical disability by center type.

**Characteristic**	**Frequency (%)**		
	**Public**	**Private**	**Total**
**SEX**			
Male	212 (52.5)	100 (49.0)	312 (51.3)
Female	192 (47.5)	104 (51.0)	296 (48.7)
Total	404 (100)	204 (100)	608(100)
**AGE**			
18-59	322 (79.3)	153 (75.7)	475 (78.1)
60≤	84 (20.7)	49 (24.3)	133 (21.9)
Total	406 (100)	202 (100)	608 (100)
Mean age	44.8 (*SD* = 14.7)	48.2 (*SD* = 15.0)	45.9 (*SD* = 14.9)
**EDUCATION**			
5< (Elementary)	12 (3.0)	8 (3.9)	20 (3.3)
5–12 (Intermediate/high school)	196 (48.3)	88 (43.4)	284 (46.6)
>12 (Upper)	198 (48.8)	107 (52.7)	305 (50.1)
Total	406 (100)	203 (100)	609 (100)
Mean years of education	13.0 (*SD* = 4.8)	13.3 (*SD* = 5.6)	13.1 (*SD* = 5.0)
**HEALTH INSURANCE COVERAGE**			
Yes	369 (91.8)	193 (97.5)	562 (93.7)
No	33 (8.2)	5 (2.5)	38 (6.3)
Total	402 (100)	198 (100)	600 (100)
**PERCEIVED SOCIAL CLASS**			
Low	113 (28.0)	26 (12.8)	139 (22.9)
Middle	289 (65.3)	144 (70.9)	408 (67.2)
High	27 (6.7)	33 (16.3)	60 (9.9)
Total	404 (100)	203 (100)	607 (100)
**ECONOMIC STATUS[RESIDENTIAL AREA PER CAPITA (m**^2^**)]**			
Under median	217 (55.2)	114 (57.0)	331 (55.8)
Upper median	176 (44.8)	86 (43.0)	262 (44.2)
Total	393 (100)	200 (100)	593 (100)
Mean of residential area per capita	38.5 (*SD* = 20.8)	44.4 (*SD* = 35.0)	40.5 (26.6)
**PERCEIVED HEALTH STATUS**			
Good health	189 (46.8)	108 (53.5)	297 (49.9)
Bad health	215 (53.2)	94 (46.5)	309 (51.0)
Total	404 (100)	202 (100)	606 (100)

As seen in Table 1, majority of people in both public and private rehabilitation centers reported themselves as belonging to middle social class. About two third of all people who reported their health status as bad, were the service users of public sector.

### Assessment of responsiveness

In all centers, a total of 126 respondents (20.9%) assessed overall responsiveness as poor, and the remainder (79.1%), perceived responsiveness as good.

**Figure 1:**
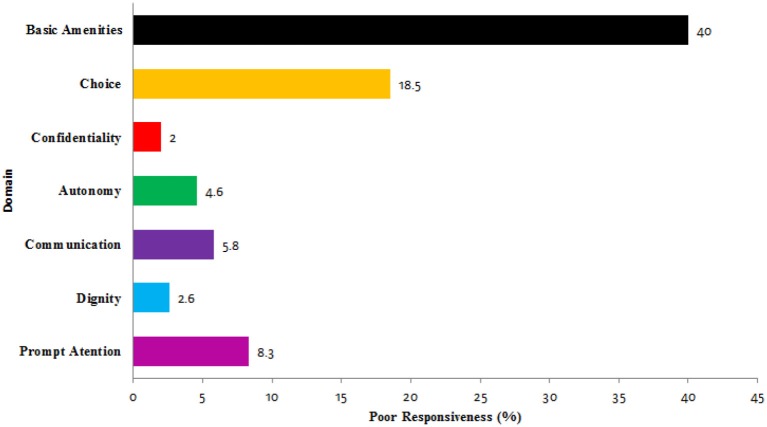
Percentage of people perceiving responsiveness as poor in each domain.

The percentage of people who reported responsiveness as poor in each domain is illustrated in Figure 1.

It can be determined from Figure 1 that confidentiality is the best performing domain, followed by dignity. Basic amenities was the poorest domain, followed by the domains of choice and prompt attention.

### Importance of the domains

**Figure 2:**
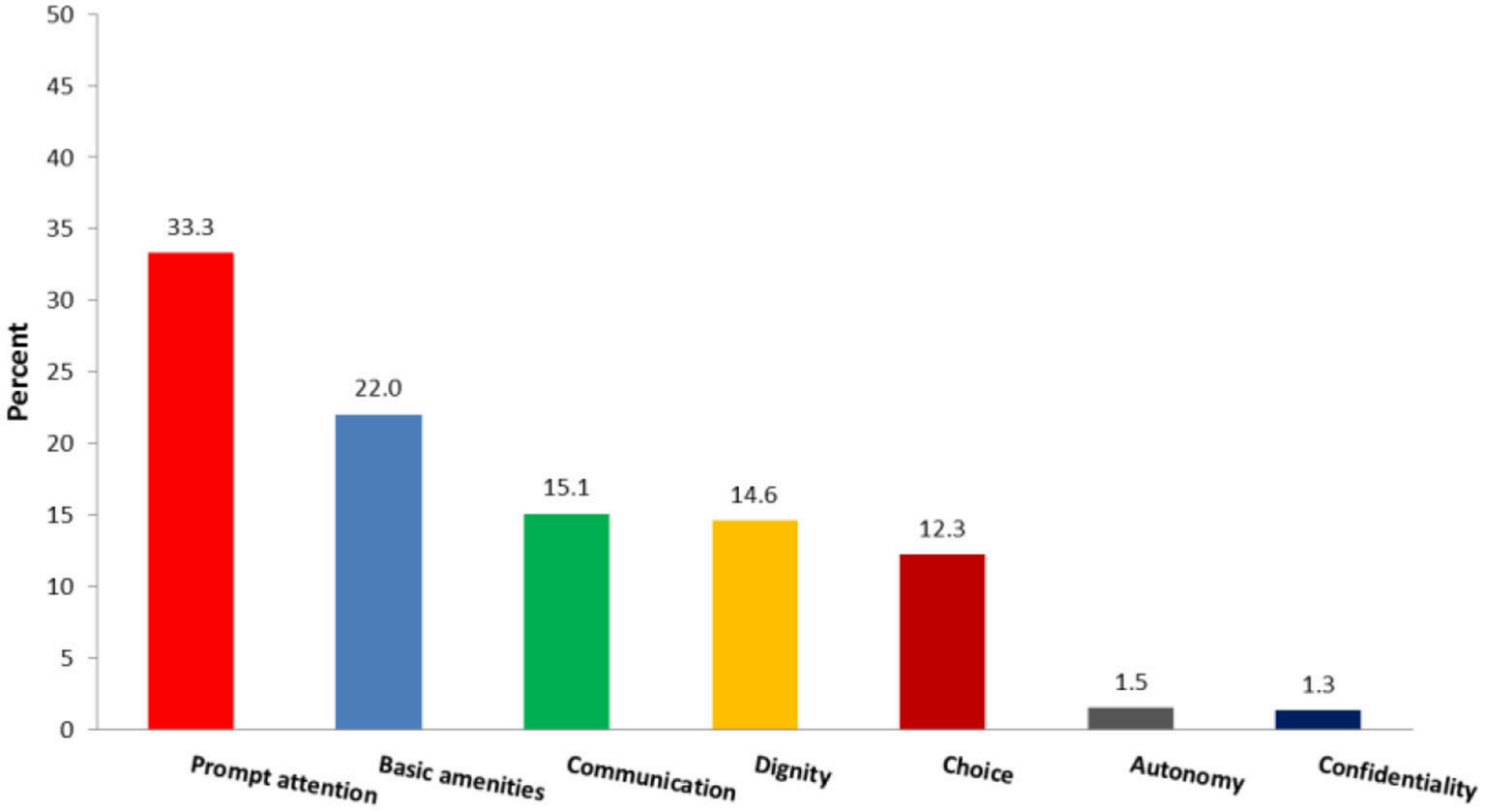
Overall responsiveness and importance of domains according to respondents' view.

The domains of responsiveness selected as the most important by respondents are shown in Figure 2.

As seen in Figure 2, prompt attention and confidentiality were the most and the least important domains, respectively.

### Responsiveness of public VS. private sector

Among participants, 22.5 and 17.9% of them rated their experience as poor in public and private comprehensive rehabilitation centers, respectively.

Comparison of responsiveness in public and private CRCs showed that people referred to public CRCs had poorer experience in the domains of communication[*x*^2^(1) = 7.95, *P* = 0.005], autonomy [*x*^2^(1) = 9.03, *P* = 0.003], and basic amenities [*x*^2^(1) = 23.76, *P* < 0.001] (Table 2).

**Table 2:** Respondents' experiences in public and private rehabilitation centers by responsiveness domains.

**Domain**	**Performance**	**Public rehabilitation centers (%)**	**Private rehabilitation centers (%)**	***x^2^* Value**	***P*-value**
Prompt attention	Good	371 (91.8)	185 (91.6)	0.011	0.91
	Poor	33 (8.2)	17 (8.4)	
Dignity	Good	390 (96.8)	198 (98.5)	1.56	0.21
	Poor	13 (3.2)	3 (1.5)	
Communication	Good	375 (92.4)	198 (98.0)	7.95	0.005^*^
	Poor	31 (7.6)	4 (2.0)	
Autonomy	Good	379 (93.6)	200 (99.0)	9.03	0.003^*^
	Poor	26 (6.4)	2 (1.0)	
Confidentiality	Good	394 (97.8)	199 (98.5)	0.387	0.53
	Poor	9 (2.2)	3 (1.5)	
Choice	Good	328 (81.0)	167 (82.7)	0.254	0.61
	Poor	77 (19.0)	35 (17.3)	
Basic amenities	Good	216 (53.2)	149 (73.8)	23.76	0.001^*^
	Poor	190 (46.8)	53 (26.2)	
Overall responsiveness	Good	307 (77.5)	165 (82.1)	1.67	0.19
	Poor	89 (22.5)	36 (17.9)	

* *Significant (P ≤ 0.05)*.

Table 2 shows that the experience of people in public and private sector was significantly different in three domains (autonomy communication, basic amenities).

**Figure 3:**
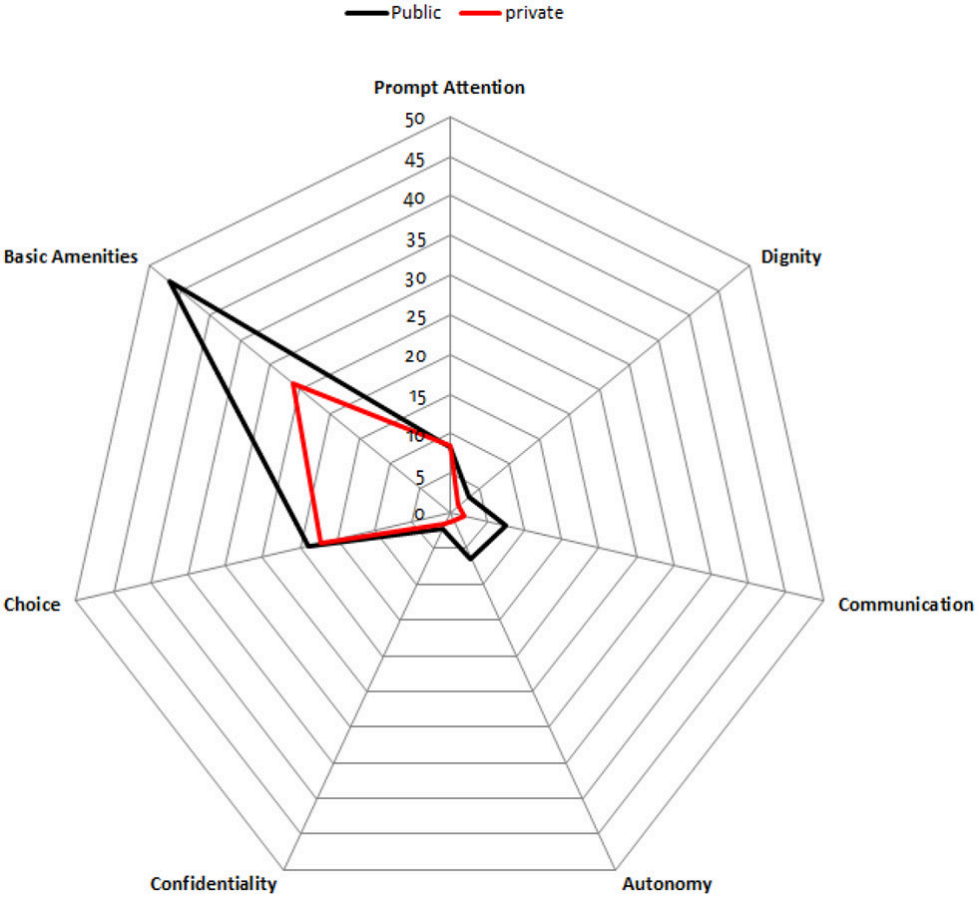
Percentages of users' rating their experience as poor by responsiveness domains in public and private CRCs.

Comparison of responsiveness domains by private and public centers is illustrated in Figure 3.

As seen in Figure 3, Based on respondents' viewpoint, performance of three domains (autonomy communication, basic amenities,) are poorer in public sector compared to private sector CRCs.

### Performance of autonomy and the gap between private and public sectors

As seen in Table 3, in lower perceived social class, lower economic status, bad health status and in age of 60 and over, poor autonomy was reported in a higher percentage.

**Table 3:** Performance of autonomy domain based on socio-demographic sub-groups' point of view by type of center.

**Characteristic**	**Autonomy performance frequency (%)**					
	**Public**		**Private**		**Total centers**	
	**Good**	**Poor**	**Good**	**Poor**	**Good**	**Poor**
**SEX**						
Male	196(92.9)	15 (7.1)	99 (100)	0 (0)	295 (95.2)	15 (4.8)
Female	183 (95.3)	9 (4.7)	101 (98.1)	2 (1.9)	284 (96.3)	11 (3.7)
**AGE**						
18-59	301 (93.8)	20 (6.2)	152 (99.3)	1 (0.7)	453 (95.6)	21 (4.4)
60≤	78 (92.9)	6 (7.1)	46 (97.9)	1 (2.1)	124 (94.7)	7 (5.3)
Mean age	45.1 (*SD* = 14.5)	40.1 (*SD* = 16.7)	47.9 (*SD* = 15.0)	55.0 (15.5)	46.1 (*SD* = 14.7)	41.2 (*SD* = 16.8)
**EDUCATION**						
5< (Elementary)	12 (100)	0 (0)	8 (100)	0 (0)	20 (100)	0 (0)
5–12 (Intermediate/high school)	184 (93.9)	12 (6.1)	84 (97.7)	2 (2.3)	268 (95.0)	14 (5.0)
>12 (Upper)	193 (92.9)	14 (7.1)	107 (100)	0 (0)	290 (95.4)	14 (4.6)
Mean years of education	13.0 (*SD* = 4.8)	13.0 (*SD* = 4.9)	13.4 (*SD* = 5.5)	8.5 (*SD* = 4.9)	13.1 (*SD* = 5.0)	12.6 (*SD* = 4.9)
**PERCEIVED SOCIAL CLASS**						
Low	102 (90.3)	11 (9.7)	25 (96.2)	1 (3.8)	127 (91.4)	12 (8.6)
Middle	249 (94.3)	15 (5.7)	143 (100)	0 (0)	392 (96.3)	15 (3.7)
High	26 (100)	0 (0)	32 (100)	0 (0)	58 (100)	0 (0)
**ECONOMIC STATUS (RESIDENTIAL AREA PER CAPITA) (m**^2^**)**						
Under median	202 (93.1)	15 (6.9)	112 (99.1)	1 (0.9)	314 (95.2)	16 (4.8)
Upper median	165 (94.3)	10 (5.7)	85 (100)	0 (0)	250 (96.2)	10 (3.8)
Mean of residential area per capita	38.4 (*SD* = 20.9)	39.5 (*SD* = 20.4)	44.5 (*SD* = 35.2)	22.5 (*SD* = 0)	40.6 (*SD* = 26.9)	38.9 (*SD* = 20.0)
**PERCEIVED HEALTH STATUS**						
Bad health	198 (92.1)	17 (7.9)	92 (97.9)	2 (2.1)	290 (93.9)	19 (6.1)
Good health	180 (95.7)	8 (4.3)	108 (100)	0 (0)	288 (97.3)	8 (2.7)

Decomposition of the gap in autonomy performance between the private and public centers is shown in Table 4.

**Table 4:** Decomposition of the gap in domain of autonomy performance by the private and public sectors.

**Variables**	**Coefficient (95% CI)**	***P-*value**
Good status (Good performance) in private	0.061 (0.037 to 0.085)	0.0001
Good status (Good performance) in public	0.005 (−0.004 to 0.015)	0.3
Differences (total gap)	0.56 (0.030 to 0.082)	0.0001
Total :Due to endowments (explained)	0.013 (0.003 to 0.023)	0.01
Age	0.002 (−0.0018 to 0.007)	0.2
Education	−0.0001(−0.0012 to 0.0009)	0.7
Perceived health status	0.002 (−0.001 to 0.001)	0.2
Economic status (Residential area Per Capita)	−0.002(−0.006 to 0.001)	0.2
Perceived social class	0.010 (0.001 to 0.020)	0.02
Total :Due to coefficients (unexplained)	0.043 (0.018 to 0.068)	0.001
Age	−0.067 (−0.154 to 0.20)	0.13
Education	−0.006 (−0.63 to 0.050)	0.8
Perceived health status	−0.038 (−0.112 to 0.036)	0.09
Economic status (Residential area Per Capita)	0.028 (−0.019 to 0.077)	0.2
Perceived social class	−0.062 (−0.172 to 0.048)	0.2
Constant	0.188 (0.140 to 0.362)	0.03

As seen in the Table 4, among the explanatory factors (age, education, perceived health status, perceived social class, economic status), perceived social class was the largest contributor in explaining inequality in autonomy performance between public and private physical rehabilitation centers (76%).

## Discussion

The current study was carried out to assess how people with physical disabilities report rehabilitation service responsiveness. To our knowledge (after an extensive literature review), there is a very limited number of studies in the field of physical rehabilitation in Iran. While being a strength of our study, our findings could therefore only be compared with studies conducted in the field of other chronic diseases.

Approximately one out of 5 people experienced poor responsiveness in the current study. Other studies on outpatient services for chronic diseases showed that in patients with mental disorders, poor responsiveness was reported by about one out of 2 service users (22), and in individuals with diabetes (25) and heart diseases (24), this rate was reported by 1 out of 3 respondents. This suggests that rehabilitation centers might have a better responsiveness rate. One possible explanation for this discrepancy is that studies of mental health responsiveness and responsiveness to patients with heart disease were implemented only in the public centers where responsiveness was rated lower overall. The other factor that should be considered is the characteristic of disease or disorder in users.

Our findings indicated that people with disabilities receiving services from rehabilitation centers in Tehran, reported their experience regarding confidentiality as the highest, followed by dignity, while they reported basic amenities as the poorest. This suggests that information related to the service users and their medical situation were not divulged, and their privacy was protected. This outcome also suggests that people with disabilities were treated respectfully in CRCs but in a physical environment that was not pleasant. Findings about best performing domains are in agreement with the results of Sajjadi et al. in people with diabetes mellitus in Tehran in 2014 (25) and of Rashidian et al., who conducted a household study about health system responsiveness in district 17 of Tehran city in 2003 (36) and of Peltzer et al. among older adults in South Africa in 2008 (37). But Wang et al. in China in their study on primary care in rural area found confidentiality as worst performing domain (38). This discrepancy could be due to different contexts as Wang studied in rural area where confidentiality may be more of concern in small population compared with large populations such as in Tehran. Results regarding the poorest performing domains supports those of Piroozi et al. in Sanandaj, a western city of Iran in 2014–2015 (27) and Torabipour et al. that investigated the responsiveness of physiotherapy clinics in Ahvaz in south of Iran in 2014 (39).

In current study, prompt attention was the most important domain from the respondents' viewpoint. This indicates that access to services in a proper waiting time was very important. This finding supports the results of Karami et al. in their study on people with heart disease in Tehran in 2012–2013 (24) as well as an investigation on responsiveness of delivery care in Thailand in 2008 (26). We also found that confidentiality was the least important domain based on respondents' viewpoint. Like vise, confidentiality was reported as the least importance in study of Mohammadi et al. in out-patient clinics of Zanjan in 2013 (40). However, Forouzan et al. found confidentiality to be one of the most important domains reported by people with mental disorders (22). The nature of disease or disorder seems to be the main factor in determining domains of higher priority.

Interestingly, our study indicated that people referred to private CRCs had a better experience than the users of public ones in terms of environment and basic amenities, communication with the health providers, and being involved in their health plans. In a study on responsiveness in Bangladesh in 2017, private rehabilitation centers were more responsive in informing and guiding the service users (41). Also, Adesanya et al. in Nigeria in 2011 found better performance of domains of dignity and prompt attention in private hospitals comparing to public sector (42). Better responsiveness of private centers has also been found in previous studies, both for outpatient and inpatient health care (19, 28, 36, 43). But Wang in rural area in China found the public sector to be more responsive in the primary care centers based on users' viewpoint. Wang reported that characteristics of service users referring to public sector was more equally distributed (38).

In this study we focused on autonomy to find how users' characteristics could explain the difference between public and private sectors. People are expecting for quality of care in domains of communication and especially basic amenities but Autonomy is more than just demanding quality of care. By participating in the decisions-making processes, patients actively exercise their fundamental rights to be involved in the health process and not to be passive about their health decisions as we see in paternalistic models (44). We found that perceived social class was the main factor which explained the gap in autonomy between the public and private rehabilitation centers. It indicates that inequalities in autonomy due to center type could be decreased if the individuals who use these services were more similar in terms of the social class that they perceive they belong to. One probable socio-economical reason could be that people perceived themselves as belonging to lower social class may refer to public centers as seen in the current study. Although responsiveness refers to non-medical, non-financial aspects of health system performance, people's orientation in the selection of public or private centers may be related to their socio-economic situation in Iran. Based on reports, percentage of private expenditure per capita out of total expenditure on health was 59.2% in Iran in 2013 and 88% of private expenditure on health estimated to be out of pocket (45). The out of pocket payments in private rehabilitation centers, may prevent people from lower socio-economic classes from accessing/using them. As a consequence, these people would turn to public rehabilitation centers. In previous study on responsiveness of public mental health centers, Forouzan et al. found that people in lower social class were more likely to report poor responsiveness (22).

Finally it should be emphasized that individual well-being is influenced by the way the person is treated (14). Understanding the experiences and expectations of service users is essential to increase the utilization of health care services, to decrease treatment dropout rates, to encourage earlier seeking of care, to be more open in interactions with health care providers and to better follow the health instructions, thus generating better health outcomes (14, 46). The way patients are treated when they interact with health systems/subsystems is important because it relates to basic human rights (47). Studies that describe and analyze how health systems are performing and how this relates to health system characteristics provide information that helps to identify the gaps in knowledge, to share the information with other countries and populations and to discuss improvements needed for better outcomes. Further investigation on people with other disabilities in terms of mental disability is recommended to assess the responsiveness of health system in the field of rehabilitation to people with disability as a vulnerable group.

### Study limitations

This study had some limitations. Non-probability sampling in Tehran was one of our limitation in current study, Therefore, generalization to other population and sub-systems should be conservative. However, the geographic location and spread of sample centers was such that we had satisfactory coverage of the service users in Tehran, both in the public and private sectors.

Another limitation was that most of the data especially on socio-demographic variables including health status were gathered based on respondents' self-report so are prone to under-reporting.

## Conclusions

Since disability is a chronic process and people with disabilities need continuous rehabilitation, the responsiveness of comprehensive rehabilitation centers is critical to successful rehabilitation.

Overall, improvement of responsiveness in domains that are of high importance from the respondents' viewpoint but are performing poorly, such as prompt attention and basic amenities, is essential. Better access to the rehabilitation centers is recommended as most of the CRCs, especially private centers, are in the north of Tehran where people with high socioeconomic status are living. Persons with disabilities should be able to choose their favorite centers and rehabilitation professionals with more freedom.

Public rehabilitation centers should provide high standards in environment, facilities and basic amenities and communicate clearly with the service users. People with disabilities using public rehabilitation centers, especially people who perceive themselves as belonging to the lower social class, should be more involved in the decision-making process regarding their health.

To be most effective, all interventions to improve responsiveness in rehabilitation centers especially public sector should involve policy-makers. Training of service providers, and informing the service users of their own rights when interacting with the health system are also important and recommended.

## Author contributions

MA conception and designing of the study, data collection, analysis of data, interpretation of data and drafting the manuscript. MRKA designing of the study, contribution in planning, implementing the study and coordinating field work, reading, and commenting the manuscript and the revised version. MM-L leading the analysis of data, interpretation of data, reading, and commenting the manuscript and the revised version. HS designing of the study, interpretation of data, reading and commenting the manuscript and the revised version. MS designing of the study, reading, and commenting the manuscript and the revised version. MN analysis of data, interpretation of data, reading and commenting the manuscript and the revised version. ASF designing of the study, analysis of data, interpretation of data and critical revision of the content of manuscript.

Additionally, Our sincere thanks and appreciation to all the comprehensive rehabilitation centers and to all the respondents who participated in this study.

### Conflict of interest statement

The authors declare that the research was conducted in the absence of any commercial or financial relationships that could be construed as a potential conflict of interest.
