# Supplementary material for: Responsiveness of Physical Rehabilitation Centers in Capital of Iran: Disparities and Related Determinants in Public and Private Sectors
Source: Front Public Health. 2018 Nov 14;6:317. doi: 10.3389/fpubh.2018.00317 (PMC6247717; doi:10.3389/fpubh.2018.00317)
Supplement: Supplementary file 1 [file Data_Sheet_1.PDF]

Table 2.2 Exact question wording, the MCSS responsiveness questions

Domain	Item number	Question wording	Type of response scale
**Prompt attention**	Q6101 Q6201 Q6303	In the last 12 months, when you wanted care, how often did you get care as soon as you wanted?	Frequency Reporting (Always - Never)
	Q6103 Q6203	Generally, how long did you have to wait before you could get the laboratory tests or examinations done?	Other Reporting (number of days)
	Q6104 Q6204 Q6304	Now, overall, how would you rate your experience of getting prompt attention at the health services in the last 12 months?	Rating (Very Good - Very Bad)
**Dignity**	Q6110 Q6210	In the last 12 months, when you sought health care, how often did doctors, nurses or other health care providers treat you with respect?	Frequency Reporting (Always - Never)
	Q6111 Q6211	In the last 12 months, how often did the office staff, such as receptionists or clerks there, treat you with respect?	Frequency Reporting (Always - Never)
	Q6112 Q6212	In the last 12 months, how often were your physical examinations and treatments done in a way that your privacy was respected?	Frequency Reporting (Always - Never)
	Q6113 Q6213 Q6305	Now, overall, how would you rate your experience of being treated with dignity at the health services in the last 12 months?	Rating (Very Good - Very Bad)
**Clear commun-ication**	Q6120 Q6220	In the last 12 months, how often did doctors, nurses or other health care providers listen carefully to you?	Frequency Reporting (Always - Never)
	Q6121 Q6221	In the last 12 months, how often did doctors, nurses or other health care providers, explain things in a way you could understand?	Frequency Reporting (Always - Never)
	Q6122 Q6222	In the last 12 months, how often did doctors, nurses, or other health care providers give you time to ask questions about your health problem or treatment?	Frequency Reporting (Always - Never)
	Q6123 Q6223 Q6306	Now, overall, how would you rate your experience of how well health care providers communicated with you in the last 12 months?	Rating (Very Good - Very Bad)
**Autonomy**	Q6131 Q6231	In the last 12 months, how often did doctors, nurses or other health care providers involve you as much as you wanted in deciding about the care, treatment or tests?	Frequency Reporting (Always - Never ever)
	Q6132 Q6232	In the last 12 months, how often did doctors, nurses or other health care providers ask your permission before starting the treatment or tests?	Frequency Reporting (Always - Never)
	Q6133 Q6233 Q6307	Now, overall, how would you rate your experience of getting involved in making decisions about your care or treatment as much as you wanted in the last 12 months?	Rating (Very Good - Very Bad)
**Confidentiality**	Q6140	In the last 12 months, how often were talks with your doctor, nurse or other health care provider	Frequency Reporting

		done privately so other people who you did not want to hear could not overhear what was said?	(Always - Never)
	Q6141<br>Q6241	In the last 12 months, how often did your doctor, nurse or other health care provider keep your personal information confidential? This means that anyone whom you did not want informed could not find out about your medical conditions.	Frequency Reporting (Always - Never)
	Q6142<br>Q6242<br>Q6308	Now, overall, how would you rate your experience of the way the health services kept information about you confidential in the last 12 months?	Rating (Very Good - Very Bad)
**Choice of health care provider**	Q6150<br>Q6250	Over the last 12 months, with the doctors, nurses and other health care providers available to you how big a problem, if any, was it to get a health care provider you were happy with?	Other Reporting (Level of problem)
	Q6151<br>Q6251	Over the last 12 months, how big a problem, if any, was it to get to use other health services other than the one you usually went to?	Other Reporting (Level of problem)
	Q6152<br>Q6309	Now, overall, how would you rate your experience of being able to use a health care provider or service of your choice over the last 12 months?	Rating (Very Good - Very Bad)
**Quality basic amenities**	Q6160	Thinking about the places you visited for health care in the last 12 months, how would you rate the basic quality of the waiting room, for example, space, seating and fresh air?	Rating (Very Good - Very Bad)
	Q6161	Thinking about the places you visited for health care over the last 12 months, how would you rate the cleanliness of the place?	Rating (Very Good - Very Bad)
	Q6162<br>Q6310	Now, overall, how would you rate the quality of the surroundings, for example, space, seating, fresh air and cleanliness of the health services you visited in the last 12 months?	Rating (Very Good - Very Bad)

PLEASE WRITE IN AT THE BOTTOM OF THE PAGE

DIGNITY[1]
- being shown respect
- having physical examinations conducted in privacy

AUTONOMY[2]
- being involved in deciding on your care or treatment if you want to
- having the provider ask your permission before starting treatments or tests

CONFIDENTIALITY OF INFORMATION[3]
- having your medical history kept confidential
- having talks with health providers done so that other people who you don't want to have hear you can't overhear you

SURROUNDINGS OR ENVIRONMENT[4]
- having enough space, seating and fresh air in the waiting room
- having a clean facility (including clean toilets)
- having healthy and edible food

CHOICE[5]
- being able to choose your doctor or nurse or other person usually providing your health care
- being able to go to another place for health care if you want to

SOCIAL SUPPORT[6]
- being allowed the provision of food and other gifts by relatives
- being allowed freedom of religious practices

PROMPT ATTENTION[7]
- having a reasonable distance and travel time from your home to the health care provider
- getting fast care in emergencies
- having short waiting times for appointments and consultations, and get tests done quickly
- having short waiting lists for non-emergency surgery

COMMUNICATION[8]
- having the provider listen to you carefully
- having the provider explain things so you can understand
- having time to ask questions

MOST IMPORTANT ________________________

LEAST IMPORTANT_______________________

Source: WHO MCSS 2000 - 2001 Brief Questionnaire
